# Defense sequestration associated with narrowing of diet and ontogenetic change to aposematic colours in the spotted lanternfly

**DOI:** 10.1038/s41598-018-34946-y

**Published:** 2018-11-15

**Authors:** Soorim Song, Shinae Kim, Sung Won Kwon, Sang-Im Lee, Piotr G. Jablonski

**Affiliations:** 1 Department of Environmental Science, Policy & Management, 130 Mulford Hall, University of California, Berkeley, CA 94720-3114 USA; 2 College of Pharmacy, Seoul National University, Seoul, 151-742 South Korea; 3 Daegu-Gyeongbuk Institute of Science and Technology School of Undergraduate Studies, Daegu, 42988 South Korea; 4 Laboratory of Behavioral Ecology and Evolution, School of Biological Sciences, Seoul National University, 08-826, Seoul, South Korea; 5 Museum and Institute of Zoology, Polish Academy of Sciences, Wilcza 64, 00-679, Warsaw, Poland

## Abstract

Bright colours in distasteful prey warn off predators, but processes associated with ontogenetic acquisition of warning colours and distasteful compounds have been studied in only a few organisms. Here, we study spotted lanternflies (*Lycorma delicatula*; Fulgoridae) that change to red colouration when they narrow their host plant preferences to primarily the tree of heaven (*Ailanthus altissima*; Simaroubaceae), which is chemically defended by quassinoids. In experiments, we showed that birds taste-avoided lanternflies collected on *Ailanthus* but not those collected on the secondary hosts. Birds also taste-avoided seeds infused with ailanthone, the main quassinoid sequestered from *Ailanthus* by lanternflies as shown through mass spectrometry analyses. Hence, the narrowing of host preferences by lanternflies synchronizes the timing of change to red colour with the acquisition of quassinoid defenses. A schematic graphical population-level model of these processes is provided. This is the first report of quassinoid sequestration by insects and the first evidence that Simaroubaceae plants provide defensive chemicals to insects. This is the first report of a fulgoroid insect sequestering identified chemical defenses. The results highlight the importance of the pan-tropical taxon Fulgoridae for evolutionary biology of complex aposematic strategies and for understanding the links between timing of defense sequestration, timing of host plant preference shifts, and timing of colour change.

## Introduction

Aposematism is an antipredatory strategy that involves chemically (or otherwise) defended unpalatable prey developing conspicuous signals to help the predators in learning the association between prey unpalatability (toxicity, unprofitability) and prey signals. While evolutionary forces shaping aposematism have been one of the central issues in evolutionary ecology^1–4^ the developmental aspects of defense sequestration and aposematic colouration during ontogeny are relatively less understood. The timing of sequestration of defenses during ontogeny can be mediated by ontogenetic changes in behavioral preferences for specific microhabitats or host plants. The accumulation of sequestered defenses during ontogeny may also be associated with ontogenetic acquisition of aposematic colour^5–9^. Despite the longstanding interest in chemical defense sequestration^10,11^ many prominently aposematic and chemically defended taxa and their host plant species remain unexamined. Brilliantly coloured phloem-sap feeders from the family of lanternflies (*Fulgoridae*) and their host plants, which include plants from the family *Simaroubaceae*^12–14^, are some of those taxa. Fulgorid phloem feeders were long suspected to follow ontogenetic changes of host plant preferences^15^. Recent quantitative data for the spotted lanternfly (*Lycorma delicatula, Hemiptera, Fulgoridae*) proved that in natural habitats these organisms ontogenetically switch from foraging on a wide range of plant species to foraging on just one primary host species^16–19^.

Apart from the research on ontogenetic change in diet breadth and density-dependent gradual colour changes in grasshoppers^20,21^, the associations between ontogenetic change in diet breadth, sequestration of defenses, and acquisition of aposematic colours are not well studied. The spotted lanternfly is a good species to study these issues. Most lanternfly species live in tropical regions and therefore their biology is relatively poorly known. However, the spotted lanternfly has recently expanded into temperate regions^19,22^, where it is intensively studied as a serious invasive pest. Therefore, knowledge about its biology quickly grows providing opportunities to examine the ecology, physiology and behaviour of Fulgoridae. Spotted lanternflies prefer a host plant from Simaroubaceae family, as do other strikingly coloured fulgorids^12–14^. The primary host of the spotted lanternfly is the globally-distributed quassinoid-containing^23-27^ tree-of heaven (*Ailanthus altissima; Simaroubaceae*). Although spotted lanternflies also preferentially feed on anthropogenic populations of grapevine, *Vitis sp*.^16–19^, this plant is unlikely to have played a major role in the natural habitats of the spotted lanternfly.

The nymphs of the first three instars are black (with white spots). The fourth instar nymphs become conspicuously red regardless of the host plant species on which they feed^17–19^, suggesting that the ontogenetic colour change is not triggered by host-plant specific chemical compounds. These insects switch from a large variety of host plants in over 18 families during the first three early nymphal stages to the quassinoid-containing tree of heaven during the red-coloured aposematic fourth instar. They largely remain on the tree of heaven during the adult stage^17–19,28^. Adult spotted lanternflies from the tree of heaven appear to be distasteful and unpalatable to birds, based on reactions of magpies, *Pica pica*^29^.We hypothesized that the behavioural shift to feeding on the quassinoid-containing primary host is linked with acquiring unpalatability from the tree of heaven.

To determine if the tree of heaven provides chemical defenses we compared the response of naïve Oriental tits (*Parus minor*) to the lanternflies collected from the tree of heaven with responses to the lanternflies collected from a rarely used secondary host plant species, the Korean willow (*Salix koreensis*). Willow chemical defense against herbivory is not based on alkaloids or quassinoids, but on phenolic glycosides^30,31^. Some of those phenolic compounds can be sequestered from willows by specialist herbivores for which willows are the primary hosts (review in Boeckler *et al*.^32^). By using a secondary host species with potentially unpalatable defensive compounds, rather than a plant species with no defensive chemicals at all, we specifically tested the hypothesis of acquisition of unpalatability from the primary rather than the secondary host plant when both hosts are chemically defended. Additionally, to evaluate the unpalatability of the spotted lanternflies to experienced insectivorous birds, we report responses of wild-caught adult tits to the taste of adult spotted lanternflies.

In order to address the idea of sequestration of quassinoids from the tree of heaven, we decided to conduct chemical analyses comparing spotted lanternflies collected from the the tree of heaven with the lanternflies from the Korean willow (*Salix koreensis*) and from another rarely used secondary host species the persimmon (*Diospyros kaki*). Persimmons are known to produce tannins but not alkaloids or quassinoids as defensive chemicals against herbivory^33^.

## Results

### Experiment 1: adult birds

**Figure 1:**
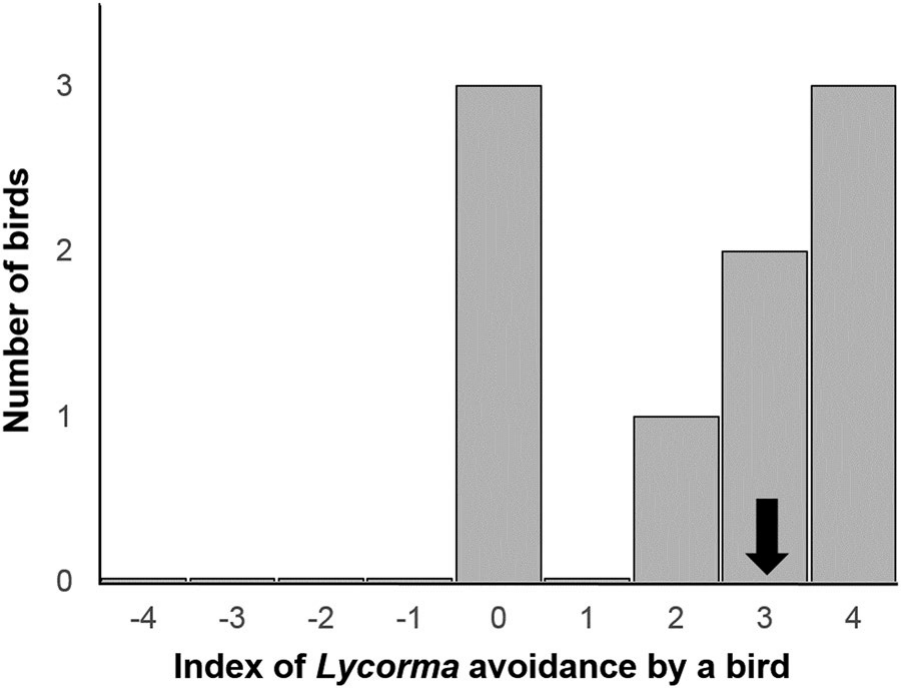
Distribution of the “*index of Lycorma avoidance*” among the nine wild-caught tits. The median (marked by arrow) is significantly larger than zero indicating that treatment food with the admixture of crushed spotted lanternflies was eaten less than the control food without admixture of lanternflies.

We tested if wild insectivorous birds rejected margarine balls (see Methods for details) containing dead squashed lanternflies from the tree of heaven (*treatment* balls) more often than pure margarine balls (*control* balls). The *control* balls were eaten or partly eaten more often than the *treatment* balls. We determined the *index of Lycorma avoidance* (Fig. 1) as the number of *control* balls eaten minus the number of *treatment* balls eaten. The median *index of Lycorma avoidance* was significantly different from zero (signed rank test for location *S* = 10.5, *n* = 9, *P* = 0.0313). This result is consistent with the expectation that the spotted lanternflies from the tree of heaven conferred some degree of unpalatability on the *treatment* balls.

At the end of each trial, all the balls were in different locations than their original positions at the onset of the experiment. This may indicate that many of the balls were picked up and dropped after taste-rejection, but we cannot exclude the possibility that balls were pushed by moving birds.

### Experiment 2: naïve birds

We tested if naïve hand raised birds, who never experienced bitter taste in any of their food items and never ate any *Lycorma* insects, differentiate between *Lycorma* of different *age* (nymph vs adult) and from different *host plant species* (*Ailanthus* vs. *Salix*) based on taste only.

#### Number of pecks

**Figure 2:**
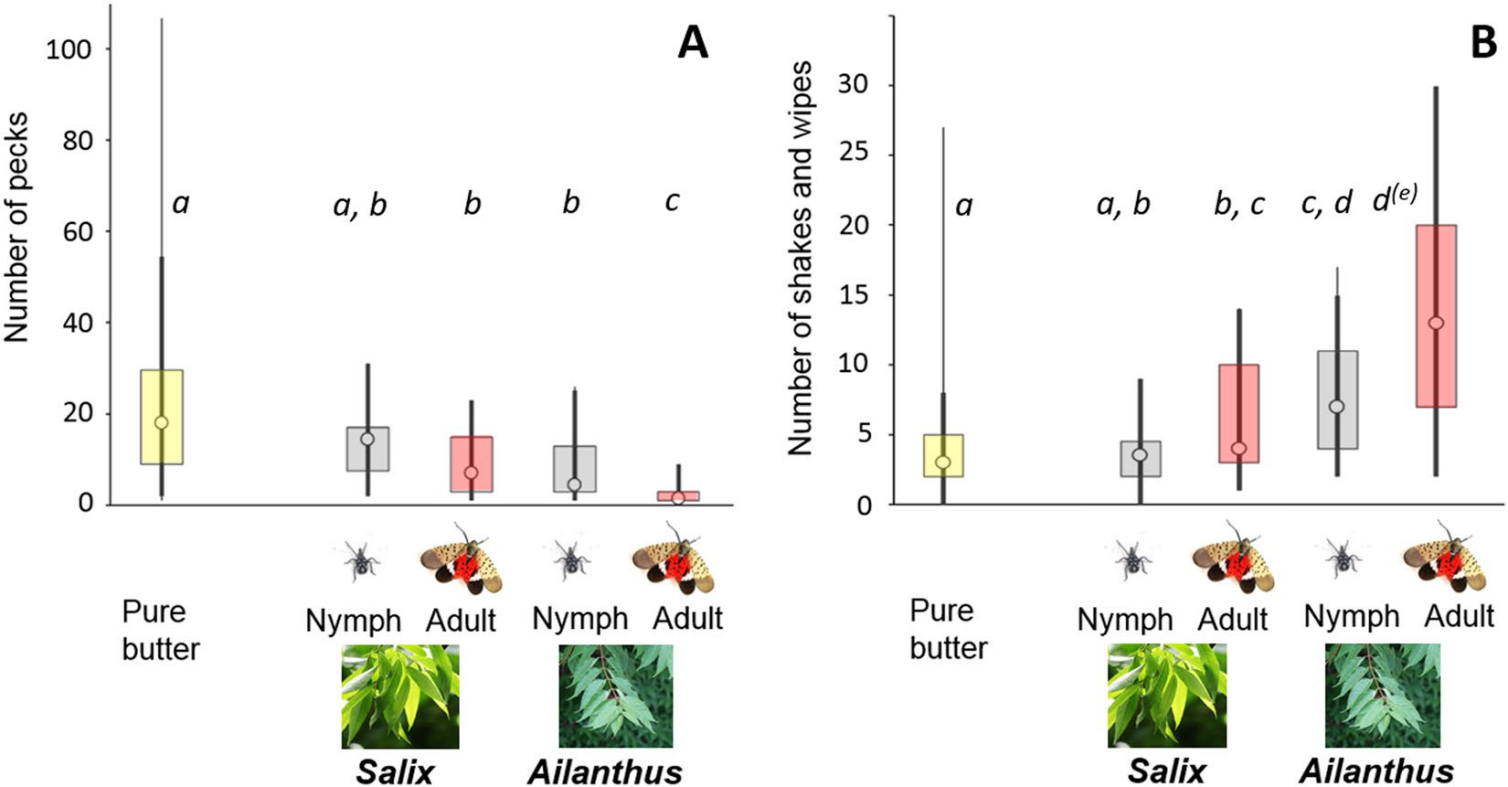
The effect of the spotted lanternflies’ (*Lycorma delicatula’s*) age (nymph *vs*. adult) and plant species on which the insects were collected (the tree of heaven [*Ailanthus*] *vs*. the Korean willow [*Salix* sp.]) on the number of pecks (**A**) by naïve hungry Oriental great tits (*Parus major minor*) at the butter mixed with insect’s tissues, and on the number of bill shakes and wipes (**B**) by the naïve birds after tasting the butter mixed with a standard amount of crushed bodies of the four types of lanternflies (nymphs from *Ailanthus*, adults from *Ailanthus*, nymphs from *Salix*, adults from *Salix*). The results from the presentations of the “Pure butter” are also presented for comparison. Thin vertical line: minimal and maximal values; thick vertical line: 5% and 95% percentiles; box: lower and upper quartiles; circle – median. The small letters indicate groups of treatments that are not significantly (*P* > *0.05*) different from each other based on the Tukey-Kramer test. In B, the “*d*^*(e)*^” indicates that if the criterion were *P* > 0.07 then the tree of heaven adults would have been a separate group because the adjusted Tukey-Kramer significance level for the comparison between tree of heaven nymphs and tree of heaven adults was *P* = 0.065.

The minimal best fit model (Table 1) contained only two fixed effects: *host* and *age*. The interaction between *host* and *age* (*P* = 0.094) was removed from the final model. However, the effect of *Lycorma’s* age on bird behaviour appeared stronger for lanternflies from *Ailanthus* than those from willows (notice a significant pairwise Tukey-Kramer comparison in Fig. 2A for *Ailanthus*). Birds performed fewer pecks at the butter with insects from the tree of heaven than at the butter with insects from the Korean willow (Fig. 2A; Fixed type III effect of *host F*_*(1,59)*_ = 13.55; *P* = 0.0005). Butter mixed with older insects received less pecks (Fixed type III effect of *age F*_*(1,59*)_ = 16.28; *P* = 0.0002) than butter mixed with younger insects (nymphs; Fig. 2A), indicating that unpalatability increases with age of insects. Pecking at butter containing adult lanternflies from the tree of heaven was especially rare, as indicated by the Tukey-Kramer multiple comparisons in Fig. 2A.

**Table 1:** Estimates for the fixed effects in the two models fitted to the data on the reactions of the Oriental great tits to the four types of butter mixed with: adult lanternflies from the tree of heaven, instar 3 lanternflies from the tree of heaven, adult lanternflies from willows, and instar 3 lanternflies from willows.

Effect	Level	Estimate (SE)	t (df)	Significance
Number of pecks:				
Intercept		2.791 (0.181)	15.46 (9)	P < 0.0001
*Host*	*Tree of heaven*	−0.773 (0.210)	−3.68 (59)	P = 0.0005
	*Willow*	0		
*Age*	*Adult*	−0.854 (0.212)	−4.04 (59)	P = 0.0002
	*Nymph*	0		
Number of shakes and wipes:				
Intercept		1.194 (0.156)	7.58 (9)	P < 0.0001
*Host*	*Tree of heaven*	0.848 (0.155)	5.46 (59)	P < 0.0001
	*Willow*	0		
*Age*	*Adult*	0.590 (0.152)	3.88 (59)	P = 0.003
	*Nymph*	0

#### Number of shakes and wipes

The fitted model (Table 1) contained only two fixed effects: *host* and *age* (their interaction was non-significant at *P* = 0.730). Birds wiped their bills and shook their heads more often after tasting the butter containing insects from the tree of heaven than the Korean willow (Fig. 2B; Fixed type III effect of *host F*_*(1,59)*_ = 29.78; P = 0.0001). Butter mixed with older insects triggered more shaking and bill-wiping than butter mixed with younger insects (Fig. 2B; Fixed type III effect of *age F*_*(1,59)*_ = 15.04; *P* = 0.0003), indicating that unpalatability increases with age. These behavioural indications of the perception of distastefulness were especially common after tasting the butter-containing adult lanternflies from the tree of heaven.

### Chemical analyses

A lack of standard chemicals, along with the absence of an adequate database at NICEM as well as the absence of raw data file, which was not provided by NICEM staff, did not allow for precise determination of chemical composition from mass spectrometry analysis of *Lycorma* from the Korean willow and from the tree of heaven. However, we could see clearly that there are differences in chemical composition between insects from *Ailanthus altissima* and those from *Salix koreensis* (see Supplementary Materials *PART 1*; compare Fig. S1A with S1C and Fig. S1B with S1D; raw outputs from analyses are in Fig. S2). These differences were much more pronounced in adult than in nymph specimens. Follow-up analysis was not possible due to the small number of samples from *Salix*.

**Figure 3:**
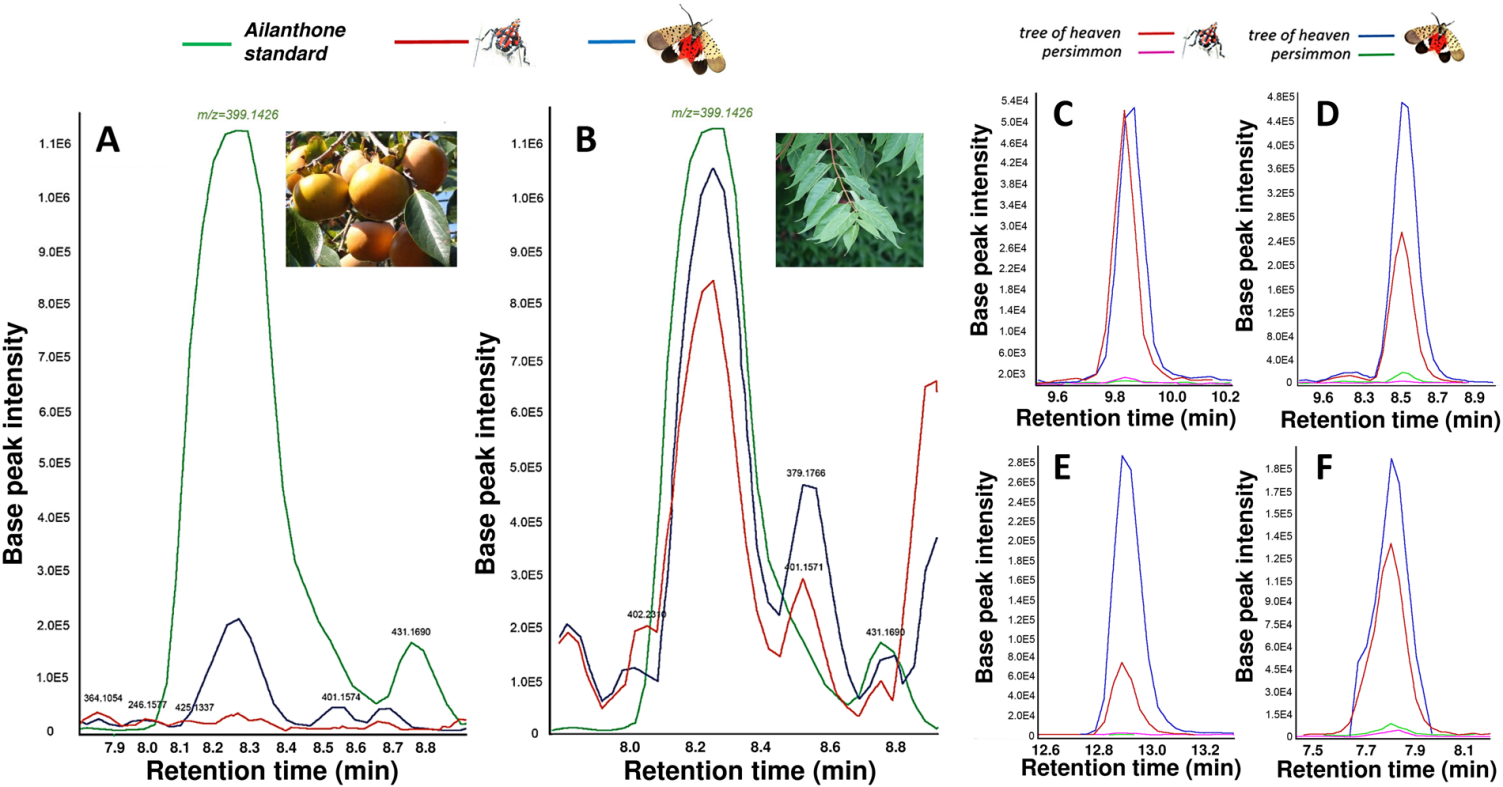
Fragments of chemical profiles of instar 4 nymphs of the spotted lanternflies collected from the persimmon tree (**A**, pink and green lines in **C,D,E,F**) and from the tree of heaven (**B**, red and blue lines in **C**,**D**,**E**,**F**). In A and B, the peaks from lanternflies (red and blue lines in A,B) are compared with the peak for the ailanthone standard (green line in A,B; m/z = 399.1426). The four peaks in C,D,E, and F are consistent with the presence in the insect bodies of the four quassinoid compounds known to occur in the tree of heaven: (**A**) amarolide (peaks at m/z within the range 356.100-365.200); (**B**) chaparrinone (peaks at m/z within range 379.158–379.177); (**C**) ailanthinone (peaks at m/z within 479.229–479.230); (**D**) shinjulactone (peaks at m/z within range 375.129–375.144). No standards of these compounds were used and therefore the identification of the four quassinoids in C–F is postulated. Notice different scales on the vertical axes indicating large differences between the compounds in the amount extracted from the spotted lanternflies. The full mass spectrometry outputs are in the Supplementary Material *PART 3*. Insert in A is by ©sphl (https://commons.wikimedia.org/wiki/File:Kaki_20041002.jpg covered by the license https://creativecommons.org/licenses/by-sa/3.0/deed.en). The remaining inserts are by P.G.Jablonski.

In the second round of chemical analyses using standard for ailanthone, we have determined that ailanthone is being accumulated in the bodies of the lanternflies feeding on the tree of heaven (Fig. 3B) but not in those feeding on the persimmon (Fig. 3A). Additionally, we found similar pattern for several peaks that match several other quassinoids known to occur in the tissues of the tree of heaven (amarolide, chaparrinone, ailanthinone, and shinjulactone; Fig. 3C–F). However, no standards were used for those quassinoids and their identities can only be postulated. The full graphical output from this mass spectrometry analysis is presented in Supplementary Materials *PART* 2.

Both analyses are consistent with the idea that the spotted lanternflies feeding on the tree of heaven gradually build up concentrations of specific chemicals that are also present in the tree of heaven (e.g. ailanthone and possibly other quassinoids) in their bodies. However, the lanternflies feeding on persimmons or willows (and possibly many other plants used by early nymphal stages) are not able to build up high concentrations of these defensive compounds.

### Experiment 3: does ailanthone deter foraging wild birds?

**Figure 4:**
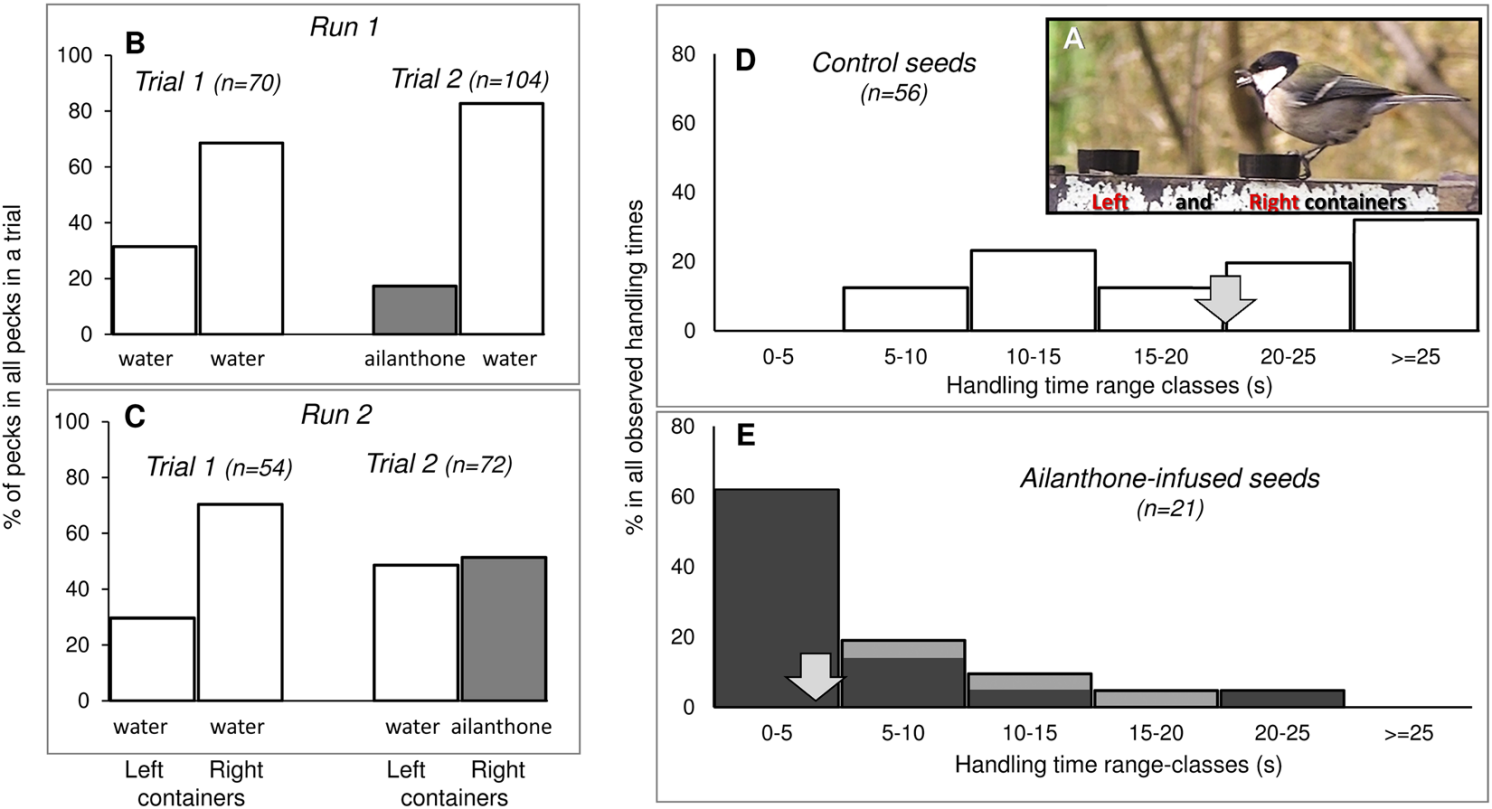
Effect of the content of sunflower seeds (ailanthone-infused seeds compared with seeds soaked in water) on their consumption by a group of 5–7 Oriental tits feeding from two adjacent containers (Left and Right container) at a winter feeder (**A**). A peck by a bird comprised picking up one seed-bit from the feeder. (**B**) Run 1 of Experiment 3: proportions of pecks (n = 70 pecks comprises 100%) at the left and right container in *Trial 1* when both containers had dry seeds previously soaked in water, and in the subsequent *Trial 2* (n = 104 pecks comprises 100%) when the left container had ailanthone-infused seeds (shaded gray). (**C**) Run 2 of Experiment 3: proportions of pecks at the left and right container in *Trial 1* (n = 54 pecks comprises 100%) when both containers had dry seeds previously soaked in water, and in the subsequent *Trial 2* (n = 72 pecks comprises 100%) when the right container had ailanthone-infused seeds (shaded gray). (**D**) distribution of handling times (s) by Oriental tits handling seeds soaked in water; (**E**) distribution of handling times by Oriental tits handling ailanthone-infused seeds. Arrows in D and E indicate median values of handling time. Dark gray shading in E indicates observations of distinct rejection by throwing away/dropping the seeds from the beak. (Photo by P.G.Jablonski).

We tested if presence of ailanthone in sunflower seeds causes avoidance reactions in a group of individually unidentifiable 5–7 Oriental tits foraging at a winter feeder comprising two plastic caps (Fig. 4A). When both containers were filled with *control* (symbol C in Fig. 4A,B) sunflower seeds (*Trial 1*), the birds tended to use the right feeder more often on both days (*Run 1* and *Run* 2) of the experiment. However, after one of the plastic caps was filled with *ailanthone-infused seeds* (symbol “A” in Fig. 4A,B) the birds avoided using this container and significantly shifted their visits to the container with *control seeds* in both runs of the experiment (*Run 1* in Fig. 4a, χ^2^ = 4.71, *df* = 1, *P* = 0.030; *Run 2* in Fig. 4B, *χ*^*2*^ = 4.62, *df* = 1, *P* = 0.032; Fisher combined *P* < 0.01) indicating that the birds avoided *ailanthone-infused seeds*. Handling time of *ailanthone-infused seeds* was shorter than handling time of *control seeds* (Mann-Whitney *Z* = 5.650, *P* < 0.00001) because the birds often rejected the *ailanthone-infused seeds* before fully consuming them. The birds almost always (dark gray shading in Fig. 4D) showed signs of discomfort such as head shaking, bill wiping, and eventually dropping/throwing the seeds away.

## Discussion

Our data suggested that feeding on the tree of heaven results in greater unpalatability compared to the secondary host, the Korean willow, regardless of the spotted lanternflies’ age. However, the especially dramatic and significant decrease in the number of pecks by birds was observed at the experimental food containing adult lanternflies from the tree of heaven. The results also show that unpalatability increased with age for both host species. It is unlikely that the unpalatability of lanternflies collected from willows was associated with a period of feeding on the tree of heaven before we collected them because we could not find any *Ailanthus* plants nearby. Therefore, we suggest that the lanternflies from the Korean willow either produced small amounts of some defensive chemicals or that they were able to sequester some defensive compounds from willows. Unlike the tree of heaven, which is defended by quassinoid and alkaloid compounds, willows are chemically defended by phenolic compounds^31,32^. This suggests that different chemicals might have been sequestered/produced by lanternflies feeding on those two different plant species.

The mass spectrometry indicated that indeed there is a difference in chemical composition between lanternflies from the Korean willow and those from the tree of heaven, and that the age-related changes in composition are more pronounced in the insects from the tree of heaven. We couldn’t reliably determine the chemical compounds responsible for these differences. However, the second mass spectrometry showed that only the lanternflies from the tree of heaven but not those from the persimmon tree accumulated the quassinoid ailanthone and possibly some other quassinoids. Consequently, insects from the quassinoid-containing tree of heaven developed higher unpalatability than insects from the secondary host plants that do not produce quassinoids. We cannot exclude the possibility that some other non-quassinoid substances sequestered from the tree of heaven might have provided the observed unpalatability. However, the combination of behavioural experiments, spectrometry analyses, and existing literature on chemical defenses in the willows and persimmons strongly suggest a causative link between sequestered quassinoids (especially ailanthone) and insect unpalatability. Future experimental studies should determine unpalatability of artificial prey soaked in quassinoids other than ailanthone, such as amarolide, chaparrinone, ailanthinone, and shinjulactone. Additionally, controlled experiments should be conducted to study if the actual concentrations of ailanthone found in lanternflies’ tissues or on their cuticle are sufficient to deter birds.

Regardless of the identity of the chemical compounds that function as sequestered defenses, the results clearly show that the increased behavioural preference for the quassinoid-providing tree of heaven happens at the time of the ontogenetic change from inconspicuous third instar to conspicuous fourth instar^18,19^. There are indications that sensitivity to some plant volatiles also increases in the third instar and is the highest in the fourth instar^34,35^. It may not be a coincidence that this shift in sensitivity to plant volatiles happens at the time of the increased preferences for the tree of heaven^16,18,19,28^. For example, linalool is one of the substances that could potentially be used to distinguish flowers of the tree of heaven, which are full of linalool, from leaves, which are poor in linalool^36^. It could also be used to distinguish young trees, with more linalool, from older trees, with less of linalool^37^. Additional studies are needed to experimentally evaluate differences in sensory and behavioural preferences for the tree-of-haven among the instars. The experimental design used by Lee *et al*.^16^ to confirm preferences for *Ailanthus* in instar 3 and in adults (instar 4 was not tested) provides a good example of how these studies could be conducted. We propose that these ontogenetic shifts in host preferences, combined with the cyclically repeated process of searching for a host plant and falling off from a plant only to start searching for a new host again (“falling-ascending cycle”^18^), lead to an increase in the proportion of individuals that feed on the preferred primary host plant. This occurs around the time when the proportion of red-coloured nymph increases in the population owing to the colour change between instar 3 and instar 4 - a change that occur regardless of host plant species^17,18^. Altogether, the compiled evidence suggests that sensory properties and behaviour of spotted lanternflies mediate the accumulation of defenses at the time when their colour changes from non-conspicuous to conspicuous.

**Figure 5:**
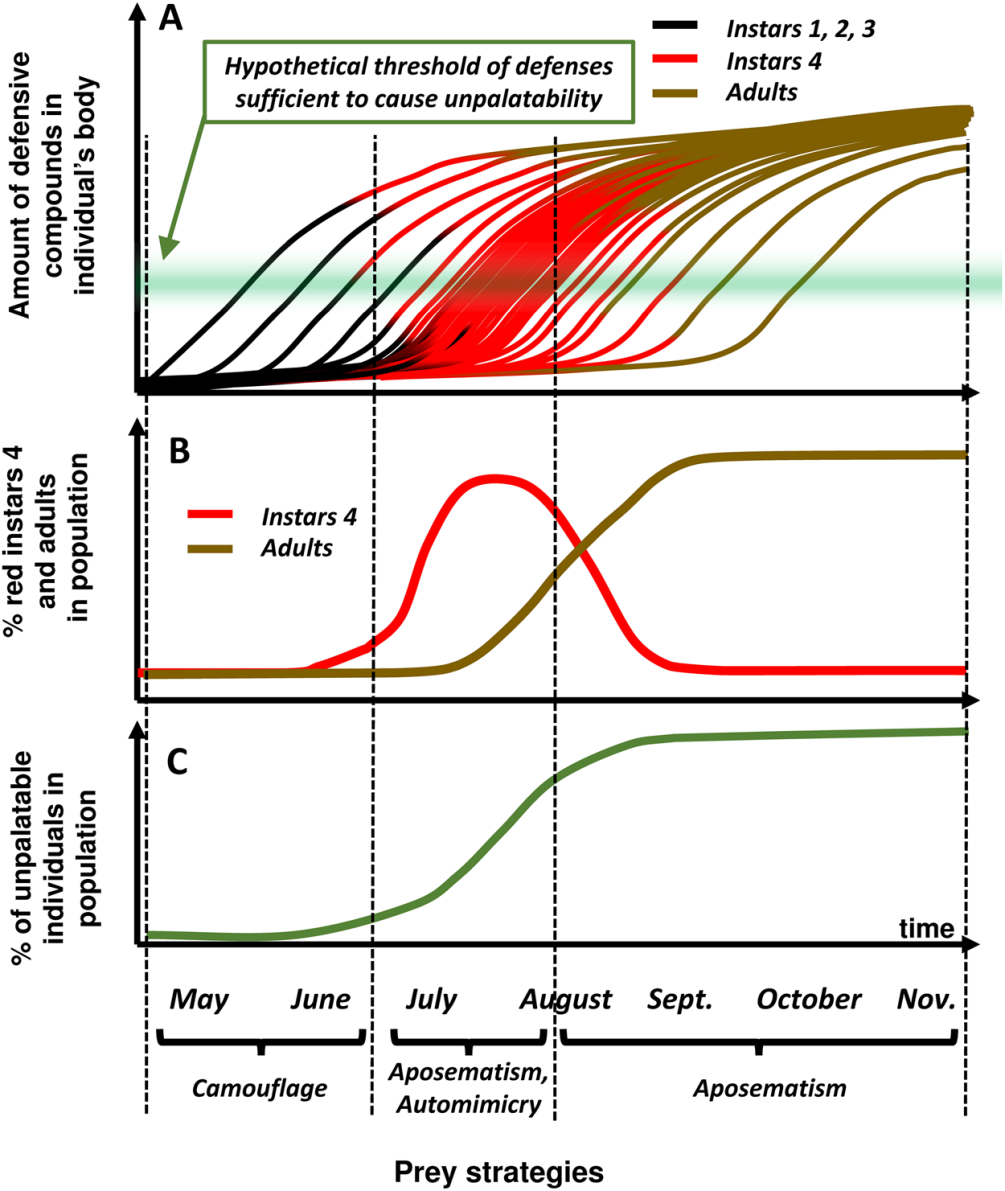
Schematic representation (graphical model) of the population-level phenomena regarding the shift of preferences for the primary host, the sequestration of chemical defenses, and the ontogenetic colour change in *Lycorma delicatula*. (**A**) - individual trajectories of accumulation of defenses in bodies of insects from a hypothetical population of the lanternflies, who preferentially switch to defense-providing tree of heaven approximately around the transition between instar 3 and instar 4. Each trajectory line changes from black (indicating instars 1, 2 and 3), to red (indicating instar 4) to brown (indicating adult), Each individual starts increasing (sequestering) chemical defenses as soon as it starts feeding on the primary host. Because this moment is different for each individual, the individual trajectories show increases of accumulated defensive compounds at different time, but most of the individuals end up on the primary host around the time of instar 4. Summarizing the individual trajectories in the whole population results in the figure representing changes of the proportion of instar 4 individuals (red) and adults (brown) in the population over time (**B**), as well as the proportion of unpalatable (defended) individuals in the population over time (**C**) assuming for simplicity that “unpalatability” happens when the amount of accumulated defensive compounds reaches a certain hypothetical threshold (shown as green-shaded horizontal bar in A).

The population level outcome of these processes should consider age variation among individuals and the inter-individual variation in the timing of the shift in feeding preferences, which results in variation of the timing of defense sequestration. This is schematically shown in a graphical model of a population of the spotted lanternfly (Fig. 5A). The population comprises a mix of individuals with different history of feeding on host plants (Fig. 5A) and with variable amounts of defensive chemicals (depending on the duration of feeding on the tree of heaven). In July, the population consists of mostly red-coloured instars 4 (Fig. 5B), many of which may not yet be properly defended due to low level of sequestered defenses (Fig. 5C; see figure caption for more explanations). Individuals with high level of chemical defense in this population are probably protected through aposematism, although their protection may be compromised by the relatively undefended red-coloured auto-mimics^38^. The auto-mimics may possibly gain some antipredatory benefits through auto-mimicry in this situation^39–41^, or because some predators may avoid prey species with high inter-individual variability in the level of defensive compounds^42^. Assuming that most individuals of the spotted lanternfly end up using the tree of heaven, the proportion of unpalatable individuals raises to near 100% through August. It remains high while the red-coloured instar 4 changes to the adult stage. Adults are protected by “facultative aposematism”^43^ involving sudden bright displays in response to predator attacks^29,44,45^.

In summary, this is the first report of sequestration of defensive quassinoids by insects and the first experimental evidence that plants in the family Simaroubaceae are the source of defensive chemicals to aposematic insects. This is also the first report of a fulgorid insect sequestering identified chemical defenses from plants. The results suggest that spotted lanternflies gradually acquire unpalatability while sequestering ailanthone and other defensive substances (possibly quassinoids) from the tree of heaven. Insects switch to using this primary host at the time when their colour ontogenetically changes to a conspicuous red. We hypothesize that there are co-evolutionary links between behavioural (ontogenetic shift to primary host), physiological (sensitivity to plant volatiles, ability to sequester quassinoids) and developmental (ontogenetic change to red colour between instar 3 and instar 4) traits as integral elements of this complex antipredatory aposematic strategy. Our results indicate that Fulgoridae is an evolutionarily unexplored taxon with diversity of striking features^46,47^ that provide new opportunities to study the complex co-evolutionary dynamics between host plants, predators and insect defenses.

## Materials and Methods

### Collection of insects

*Lycorma delicatula* nymphs and adults were collected in August-October 2011, May-September 2012 and July-August 2015 at several locations in Seoul. For the experiments with adult birds we used adult insects collected from tree of heaven in 2011. For the experiments with naïve birds, we used specimens from *Ailanthus altissima* collected at Incheon elementary school, Naksongdae, Seoul and specimens from *Salix koreensis* saplings in Saetgang Park, Yeongdeungpo-gu. In 2015, specimens for mass spectrometry were collected from tree of heaven at SNU campus and from persimmon tree, *Diospyros kaki*, between the Hoam Faculty House and Science Park in Naksongdae, Seoul. Collected insects were transported in net bags and stored in a freezer in tightly closed plastic zip-lock bags for later use.

All methods were carried out in accordance with the regulations of the Seoul National University, and in accordance with the law of Korea. All experiments were approved by the Seoul National University’s IACUC permit nr SNU-130621-6 and the research permit from the Gwanak-gu office in Seoul.

### Experiment 1: adult birds

To determine if the taste of *Lycorma delicatula* was associated with avoidance by avian predators, we examined avian responses to food items containing crushed spotted lanternflies from *Ailanthus* in the absence of visual cues suggesting its presence. We used varied tits *(Poecile varius*) and Oriental tits (*Parus minor*), which are common insectivorous birds in the area of study. Their foraging behaviour makes them likely predators of *Lycorma*. In January/February 2012 we tested 8 varied tits and 1 Oriental tit. Each bird was captured in a mist net at a feeder in the Gwanak Mt. area and put in a cage (40 × 50 × 40 cm) with food and water. On the next morning the seeds were taken away and 8 balls made from margarine (8–10 mm in diameter) were provided. Four of the balls served as *control* without insects added. Four were the *treatment* balls, where we crushed an adult *Lycorma* specimen and mixed it with margarine. The surface of *control* and *treatment* balls appeared identical when inspected visually. Initial observation of the birds confirmed that they were able to taste-reject a ball after touching it with the tip of the tongue. We let the birds stay in the cages undisturbed in a closed room for 3 hours, then counted the number of fully or partly eaten balls. To determine whether birds tasted the material, we looked for balls moved away from their original positions. The birds were released at the site of capture one hour after the experiment. These procedures were conducted in accordance with the IACUC permit nr SNU-130621-6.

We determined the *index of Lycorma avoidance* by birds: the number of *treatment* balls eaten and partly eaten minus the number of *control* balls eaten or partly eaten. The null expectation was that this index should not differ significantly from zero if there is no preferential avoidance or consumption of the balls containing crushed lanternflies.

### Experiment 2: naïve birds

#### Collection and care of young birds

In June 2012, eleven 8–10 days old Oriental tit (*Parus minor*) nestlings were taken from three nest boxes located in our study population at SNU campus and Mount Gwanak. The nestlings were hand-raised on a diet based on insects and fatty seeds. Food and water was provided *ad libitum*, and the birds were maintained in indoor cages. They were handled regularly and trained to accept food from human hands. Nine males and two females were used in the trial(sex determined through the standard DNA-base method). All birds were reared to adulthood and were subsequently released. The procedures were conducted in accordance with the IACUC permit SNU-130621-6 and with the local government permit from the city of Seoul administration.

#### Experimental treatments with naïve birds

We observed reactions of birds after tasting a small amount of butter provided on a tip of a toothpick. Unsalted butter was purchased in July 2012 and was divided into five blocks weighing 50 g each. One block was used as *control*. The remaining four were mixed with ground *Lycorma* and used in *treatment* tests. We used two *age* categories: *nymph* (3rd instar nymph) and *adult*. Within each of these two categories the insects were divided into two classes according to the *host plant species* on which they were found: *willow* (*Salix koreensis*) and *tree of heaven* (*Ailanthus altissima*). 5 g of the fresh mass in each age/host combination were crushed in a mortar. Each sample was mixed with a block of unsalted butter with a spatula and was offered to the birds.

#### Scoring the birds’ responses

Birds were trained to peck at the butter on the tip of a toothpick prior to the experiment. All eleven birds responded to pure unsalted butter by pecking. Birds were starved for two hours prior to each trial. The mixture of unsalted butter and insects was presented to the birds, and the response of the bird was recorded using a voice recorder for later transcription. The observer counted the number of pecks at the butter and the number of head-shakes and bill-wipes immediately following the pecking. The observer stopped recording when the bird moved away from the experimenter for more than a minute. To minimize the effect of association between butter and unpleasant taste, untreated pure butter (*control*) was repeatedly given to the birds for at least one day until the next treatment test was performed. The number of pecks and the subsequent head-shakes and bill-wipes were also noted during the presentations of the pure butter. Each individual bird was tested 1–5 times for each of the four experimental treatments and 11–17 times for the pure butter tests applied between the treatments. The order of treatments was random and different for each bird.

### Statistical analysis of experiments

We evaluated the null hypothesis that the *index of Lycorma avoidance* for the nine birds tested in Experiment 1 did not differ from zero. We used the median sign test, which does not require the distribution to be symmetric. We analysed two separate response variables from experiment 2: the count of pecks on the insect-butter mixture and the sum of the count of head-shaking plus bill wiping behaviours after tasting the mixture. We assumed that the more desirable food received more pecks and was followed by less shakes and wipes. We used generalized linear mixed models to determine the effect of the *host* species (willow vs tree of heaven), lanternfly’s *age* (nymph vs adult), and their interaction on birds’ responses. Bird identity was entered as random effect. Negative binomial distribution with log link was chosen to adequately account for residual over dispersion in the data. Finally, to compare the bird responses in the four *treatments* to their responses to the pure butter (*control*) we used the Tukey-Kramer multiple comparison method among control and each of the four treatments. This method accounts for unequal sample sizes between the control and treatment trials. Analyses were performed using the lme4 package (version 1.1-9) (Bates *et al*. 2015) in R 3.2.4 and the procedures GLIMMIX, LSMEANS and UNIVARIATE in SAS ver 9.3.

### Chemical analyses

Preliminary analysis confirmed that in our population of the spotted lanternflies, unlike in the Chinese population studied by Xue & Yuan^48^, yohimbine or ajmalicine were not detected in their bodies (See Supplementary Materials *PART 6*). These alkaloids are known to function as insect defenses sequestered from plants but are not reported in the tree of heaven or any common secondary hosts in our population^29,30^. After we ruled out these compounds, we conducted two different high resolution mass spectrometry analyses to determine the effect of tree of heaven as a host species upon the chemical composition of the insect body.

#### LCQ mass spectrometry

For the comparison between insects from willows and those from the tree of heaven, 0.1 g dry weight of *L. delicatula* specimens was crushed with a spatula. 1 mL MeOH was added and the mixture was sonicated for 30 minutes. Specimens were classified according to developmental stage (nymph instars 3, 4 and adults) and the host plant species (the tree of heaven and the Korean willow).

Prepared sample was analysed by LCQ mass spectrometry by NICEM at Seoul National University. An Acquity HSS T3 column with 1.8 μm particles with Solvent A (Water, 0.2% formic acid) and solvent B (98% acetonitrile, 2% water with 0.2% formic acid) were used. Separation was carried out under the following condition: 5 min 90% A, 2.5 min 90–0% A, 10 min 0% A. then a fast return in 0.1 min to 100% A and 2 min for column equilibration. The flow rate was increased from 350 μL/min. Mass spectrometry was carried out with a Thermo Finnigan LCQ Deca XP Plus mass spectrometer operated in positive and negative mode. A full scan (*m/z* 50–2000) at 30,000 and 60,000 resolving power was conducted. Ions were generated through probe electrospray ionization (PESI). For collision induced dissociation (CID), normalized collision energy of 25% was used.

#### Liquid chromatography triple quadrupole mass spectrometry

We conducted a second set of mass spectrometry analyses at the College of Pharmacy, Seoul National University. Due to the lack of samples available from *Salix koreensis* saplings, nymphs instar 4 and adults were newly collected from *Ailanthus altissima* and *Diospyros kaki* and prepared in a procedure identical to the first spectrometry analyses, with two modifications: instead of sonication the insects were extracted with MeOH with crushing and vortexing for five minutes each and filtered by 0.2 µm PTFE syringe filter. Ailanthone, the main quassinoid present in *Ailanthus altissima* tissues, was used as a standard. The identity of the other compounds was proposed based on their mass per charge ratio (*m/z*) and unique fragmentation patterns. The analysis was performed using an Agilent HPLC system Equipped with Acquity™ UPLC column (BEH C18, 1.7 μm; 2.1 mm × 100 mm) and Agilent Q-ToF 6530 mass system (Agilent, USA), both with positive ion modes. 5 μL of each sample was injected and separated by the following gradient method: Solvent A (water + 0.1% formic acid) and Solvent B (acetonitrile + 0.1% formic acid): 0 min 100% A; 5 min 70% A; 15 min 30% A; 25 min 20% A; and 27~ min 0% A. The flow rate was 0.15 mL/min. The column temperature is maintained 40 °C. Mass spectrometry parameters were as follows: scan time, 100 scans/sec; cell accelerator voltage, 7 V; fragmentor, 125 V; gas temperature, 300 °C; gas flow, 10 L/min; and capillary voltage, 4 kV. The mass range was from 50 to 1500 m/z. HPLC grade acetonitrile, methanol, and water were obtained from J.T. Baker (Avantor, Philipsburg, NJ, USA). Polytetrafluoroethylene (PTFE) syringe filters with 0.20 µm pore were obtained from Advantec (Tokyo, Japan). Ailanthone standard (>98%) was purchased from Sigma Aldrich (UK).

### Experiment 3: does ailanthone deter foraging wild birds?

Chemical analyses pointed to ailanthone as one of the hypothetical chemical defenses sequestered from the tree of heaven. Therefore, we tested if presence of ailanthone in food is sufficient to cause avoidance reactions in foraging birds. We presented wild birds with crushed sunflower seeds that were dried after being soaked for 1 day in either in water (*control seeds*) or in aqueous 0.1% solution of ailanthone (*ailanthone-infused seeds*). Ailanthone was ordered from EnsolBio Sciences, Korea (Catalog nr. ES076-A). The “*ailanthone-infused seeds*” were detectably bitter to humans (as were specimens of *Lycorma* from the tree of heaven). The feeder consisted of two round black plastic containers, ~2 cm in diameter, and ~1 cm in depth located about 10 cm apart from each other (Fig. 4E). We conducted two runs of this experiment (on March 12^th^, and 16^th^, 2018). In each run, we conducted two 40-minute trials. In the *Trial 1* we presented birds with *control seeds* in two containers followed by *Trial 2* conducted after replacing one of the containers with a container filled with *ailanthone-infused seeds*, and refilling the second container with *control seeds* so that both containers had the same amount of seeds. In *Trial 2* of the first run the *ailanthone-infused seeds* were put in the left container and in the *Trial 2* of the second run the *ailanthone-infused seeds* were put in the right container. The feeder that was regularly visited by 5–7 Oriental tits that were individually un-identifiable. We focused on single events of taking seeds from the container. We also measured handling durations for those instances when the bird flew out to a nearby branch and remained in the view of the observer. We recorded any signs of discomfort or seed rejection by birds. Obviously, the statistical analyses from one group cannot be treated as generalizations about the local population of Oriental tits. But, the results can provide conclusions about a population of seed pick-up events and handling events performed by this particular group of birds at a specific feeder location. We have no reasons to believe that the group composition changed during the 80 minutes of each Run because tit flocks hold winter territories leading to stable composition of birds at a feeder within a group territory. For analysis of frequencies, we used Chi-square test separately for the first and second Run, followed by Fisher combined test. For the analysis of handling durations we use Mann-Whitney test.

## Electronic supplementary material

Supplementary Information

## Data Availability

The datasets generated during and/or analysed during the current study are available from the corresponding author on reasonable request.
