# Supplementary material for: Defense sequestration associated with narrowing of diet and ontogenetic change to aposematic colours in the spotted lanternfly
Source: Sci Rep. 2018 Nov 15;8:16831. doi: 10.1038/s41598-018-34946-y (PMC6237927; doi:10.1038/s41598-018-34946-y)
Supplement: Supplementary file 1 — Supplementary Information [file 41598_2018_34946_MOESM1_ESM.pdf]

SUPPLEMENTARY MATERIAL

Defense sequestration associated with narrowing of diet and ontogenetic change to aposematic colors in the spotted lanternfly.

Soorim Song[1], Shinae Kim[2], Sung Won Kwon[2], Sang Im Lee[*3,4], Piotr G. Jablonski[*4,5]

[*]Corresponding authors: S.I.Lee & P.G. Jablonski;
Corresponding email: **sangim@dgist.ac.kr** and also: snulbee@behecolpiotrsangim.org

Addresses
[1] Department of Environmental Science, Policy & Management, 130 Mulford Hall, University of California, Berkeley, CA 94720-3114

[2] College of Pharmacy, Seoul National University, Seoul 151-742, South Korea.

[3] Daegu-Gyeongbuk Institute of Science and Technology School of Undergraduate Studies, Daegu 42988, South Korea.

[4] Laboratory of Behavioral Ecology and Evolution, School of Biological Sciences, Seoul National University, 08-826 Seoul, South Korea

[5] Museum and Institute of Zoology, Polish Academy of Sciences, Wilcza 64, 00-679 Warsaw, Poland

running headline: OCC in lanternflies and sequestration of defenses

PART 1) Results of first set of mass spectrometry analyses ("LCQ mass spectrometry ")

Here we present the summary (Fig. S1) and the original mass spectrometry outputs (Fig. S2.). A1, A2, A3 in Fig. S2 correspond to Fig.S1 A; B1, B2, B3 in Fig. S2 correspond to Fig. S1. B; C1, C2, C3 in Fig. S2 correspond to Fig. S1 C; D1, D2, D3 in Fig. S2 correspond to Fig. S1 D.

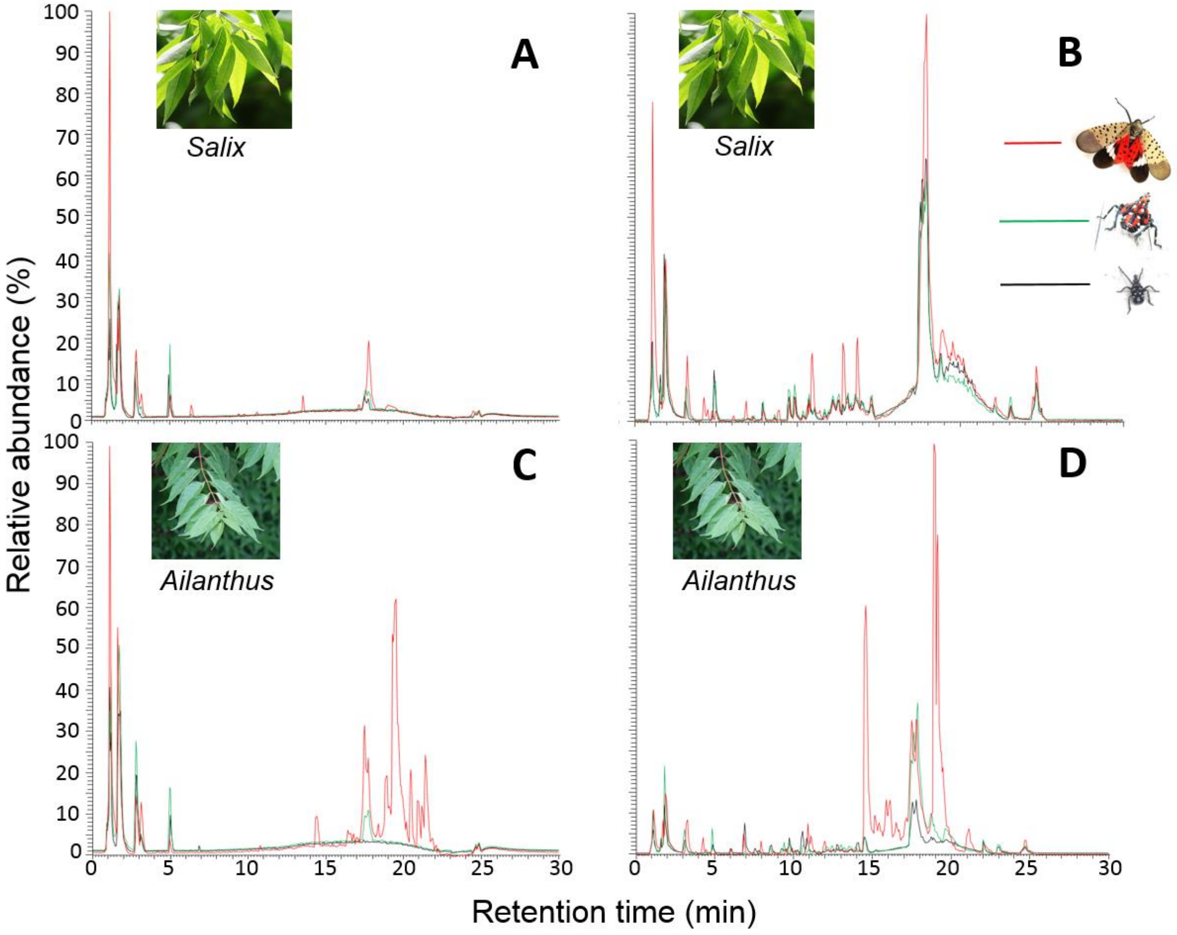

Fig. S1. Modified mass spectrometry profiles from the positive mode (A, C) and the negative mode (B, D) of mass spectrometry analyses of insects from the Korean willow (A, B) and from the tree of heaven (C, D). Only an approximate comparison in terms of relative abundance can be made cautiously between coloured lines within each of the panels. It is only a very approximate comparison: the peak heights for nymph 3 and nymph 4 have been adjusted down such that the bottom lines of the spectrograms (the basal "noise lines") overlap for all the three age stages, as if the amount of compounds responsible for these basal lines were unchanged between age stages. The original, non-manipulated figures showing relative abundances obtained from the mass spectrometry analyses (relative to the highest peak in each analysis separately) are in Fig. S2.

The next 7 pages contain Fig. S2 and its caption

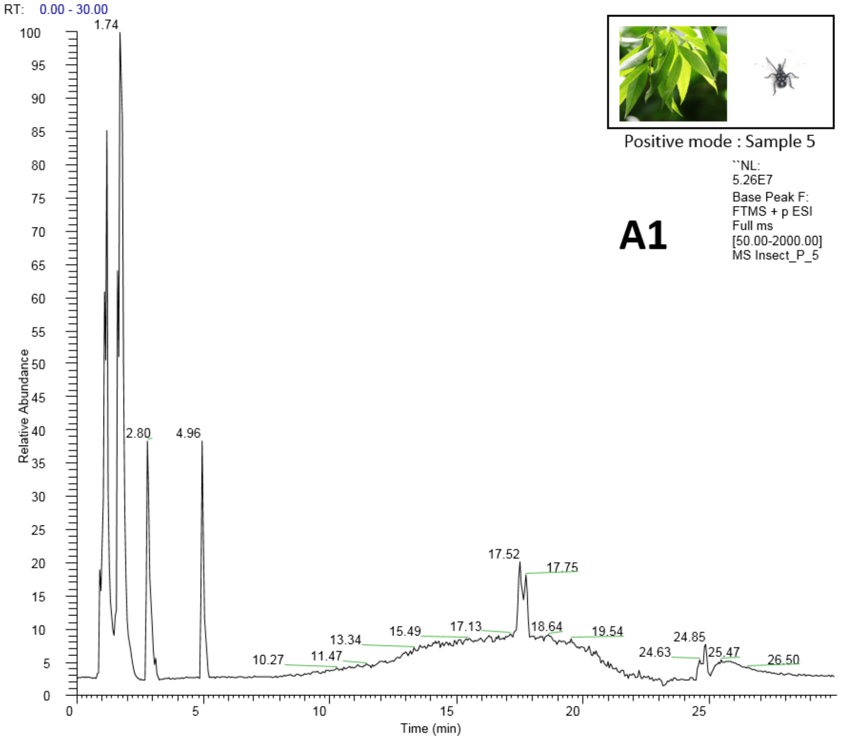
RT: 0.00 - 30.00
Relative Abundance
Time (min)
1.74
2.80
4.96
10.27
11.47
13.34
15.49
17.13
17.52
17.75
18.64
19.54
24.63
24.85
25.47
26.50
Positive mode : Sample 5
NL:
5.26E7
Base Peak F:
FTMS + p ESI
Full ms
[50.00-2000.00]
MS Insect_P_5
A1

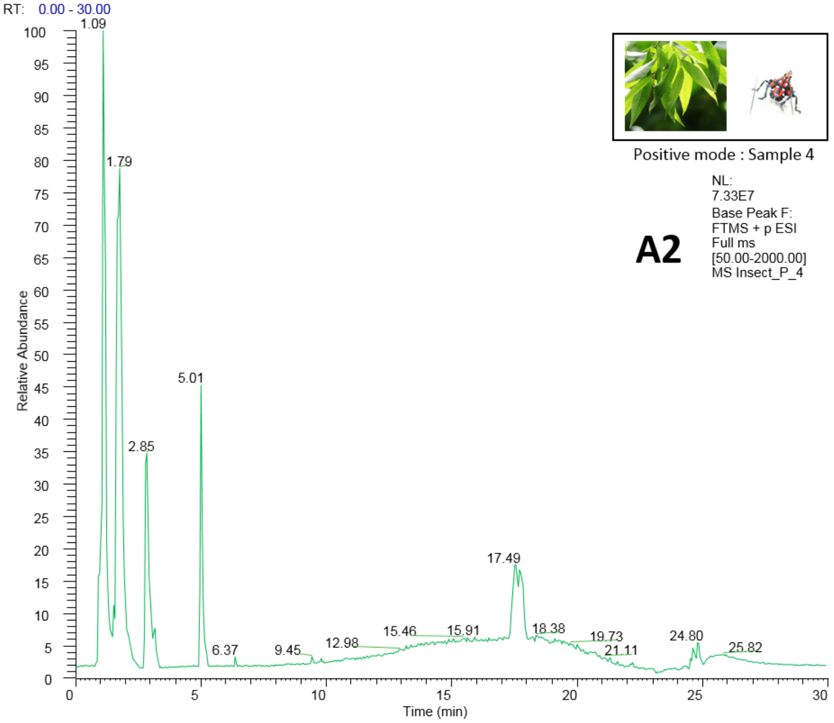
RT: 0.00 - 30.00
Relative Abundance
Time (min)
1.09
1.79
2.85
5.01
6.37
9.45
12.98
15.46
15.91
17.49
18.38
19.73
21.11
24.80
25.82
Positive mode : Sample 4
NL:
7.33E7
Base Peak F:
FTMS + p ESI
Full ms
[50.00-2000.00]
MS Insect_P_4
A2

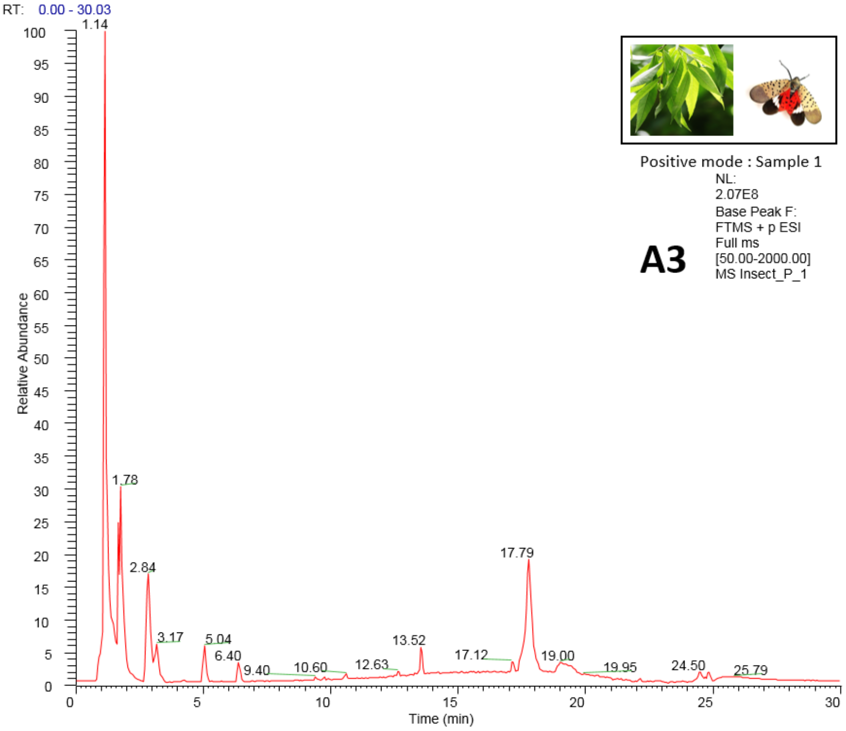
RT: 0.00 - 30.03
Relative Abundance
Time (min)
1.14
1.78
2.84
3.17
5.04
6.40
9.40
10.60
12.63
13.52
17.12
17.79
19.00
19.95
24.50
25.79
Positive mode : Sample 1
NL:
2.07E8
Base Peak F:
FTMS + p ESI
Full ms
[50.00-2000.00]
MS Insect_P_1
A3

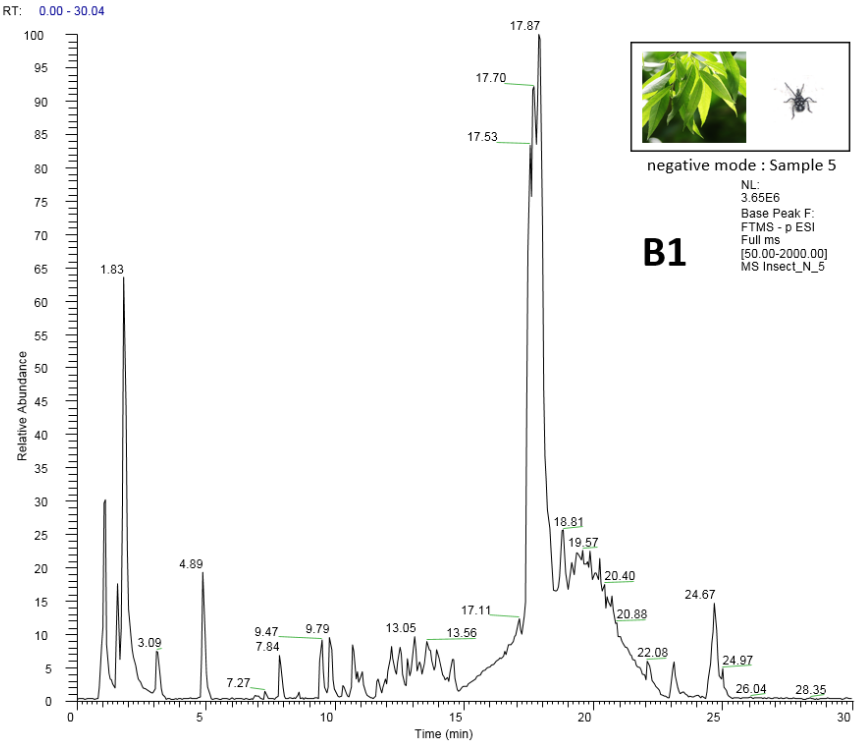
RT: 0.00 - 30.04
Relative Abundance
Time (min)
1.83
3.09
4.89
7.27
7.84
9.47
9.79
13.05
13.56
17.11
17.53
17.70
17.87
18.81
19.57
20.40
20.88
22.08
24.67
24.97
26.04
28.35
negative mode : Sample 5
NL:
3.65E6
Base Peak F:
FTMS - p ESI
Full ms
[50.00-2000.00]
MS Insect_N_5
B1

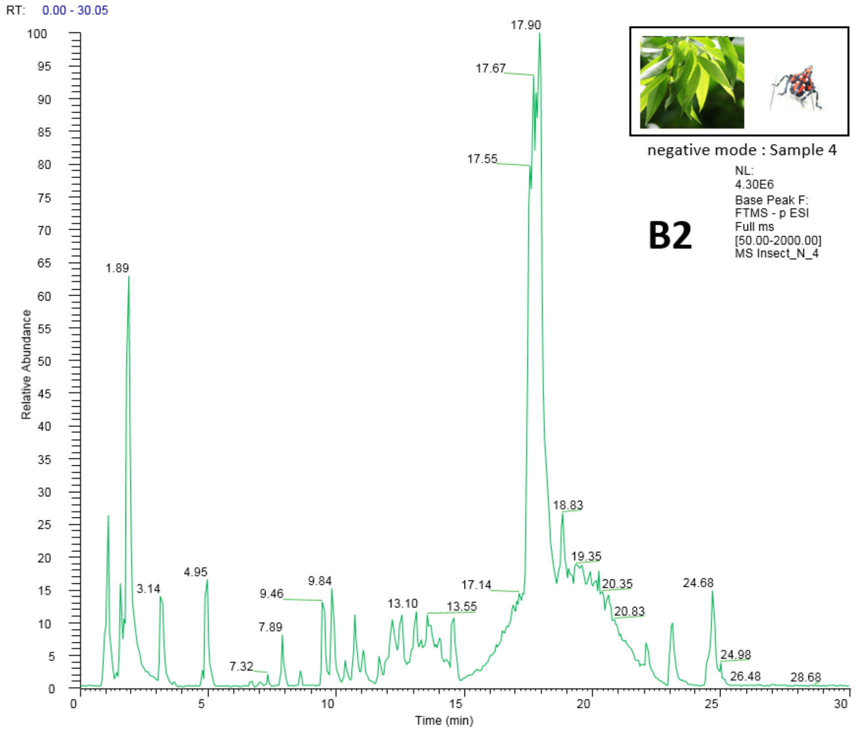
RT: 0.00 - 30.05
Relative Abundance
Time (min)
17.90
17.67
17.55
1.89
18.83
19.35
20.35
20.83
4.95
3.14
9.46
9.84
7.89
7.32
13.10
13.55
17.14
24.68
24.98
26.48
28.68
negative mode : Sample 4
NL:
4.30E6
Base Peak F:
FTMS - p ESI
Full ms
[50.00-2000.00]
MS Insect_N_4
B2

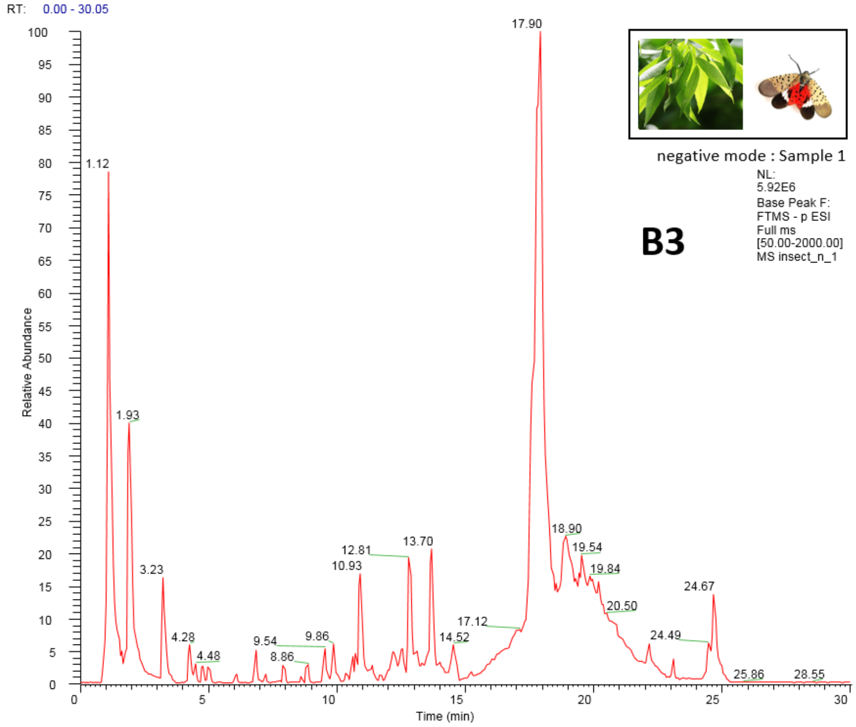
RT: 0.00 - 30.05
Relative Abundance
Time (min)
17.90
1.12
1.93
3.23
4.28
4.48
9.54
8.86
9.86
12.81
10.93
13.70
14.52
17.12
18.90
19.54
19.84
20.50
24.67
24.49
25.86
28.55
negative mode : Sample 1
NL:
5.92E6
Base Peak F:
FTMS - p ESI
Full ms
[50.00-2000.00]
MS insect_n_1
B3

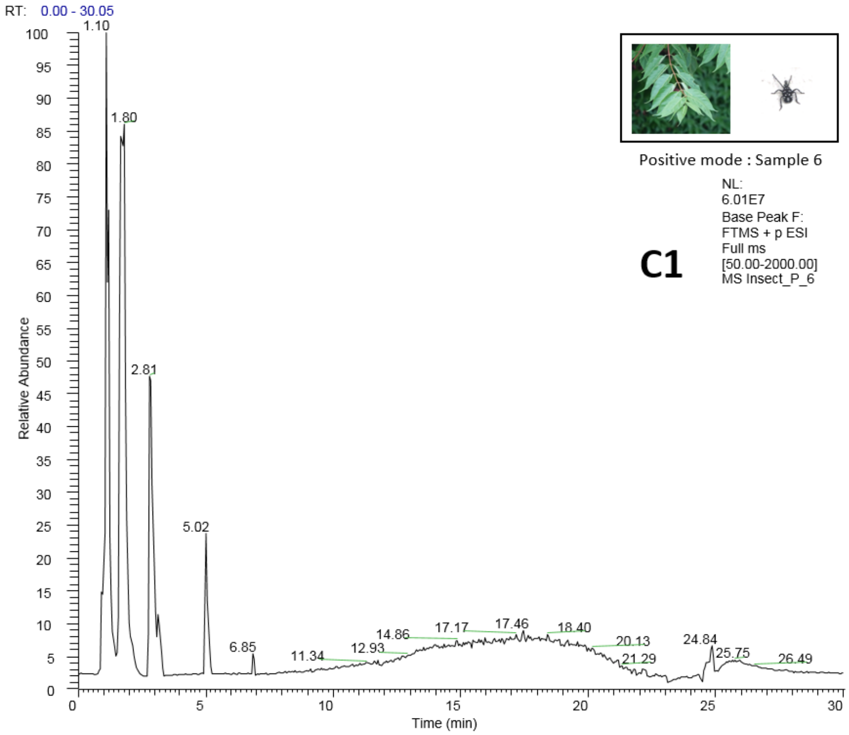
RT: 0.00 - 30.05
Positive mode : Sample 6
NL:
6.01E7
Base Peak F:
FTMS + p ESI
Full ms
[50.00-2000.00]
MS Insect_P_6
C1
Relative Abundance
Time (min)
1.10
1.80
2.81
5.02
6.85
11.34
12.93
14.86
17.17
17.46
18.40
20.13
21.29
24.84
25.75
26.49

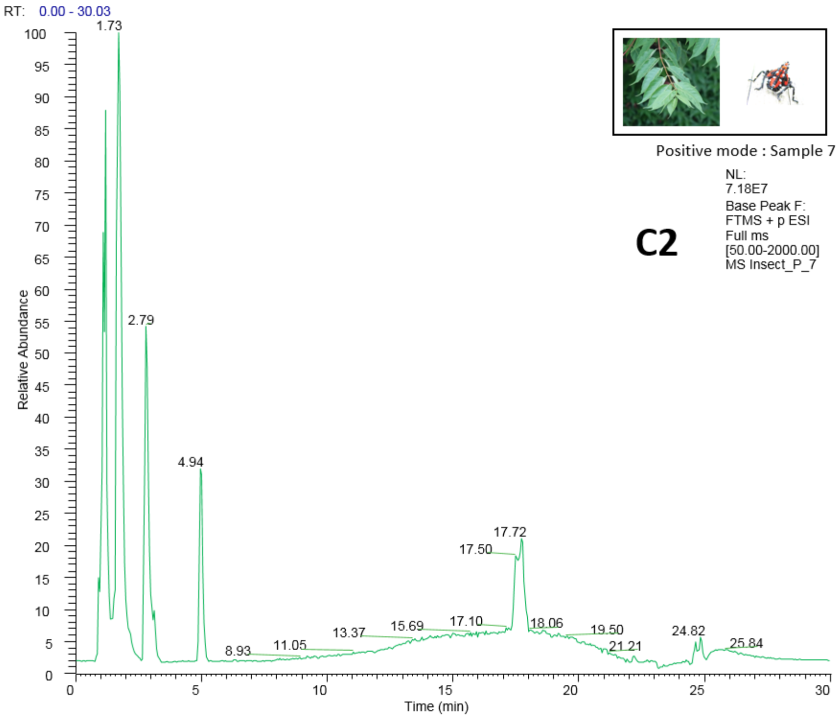
RT: 0.00 - 30.03
Positive mode : Sample 7
NL:
7.18E7
Base Peak F:
FTMS + p ESI
Full ms
[50.00-2000.00]
MS Insect_P_7
C2
Relative Abundance
Time (min)
1.73
2.79
4.94
8.93
11.05
13.37
15.69
17.10
17.50
17.72
18.06
19.50
21.21
24.82
25.84

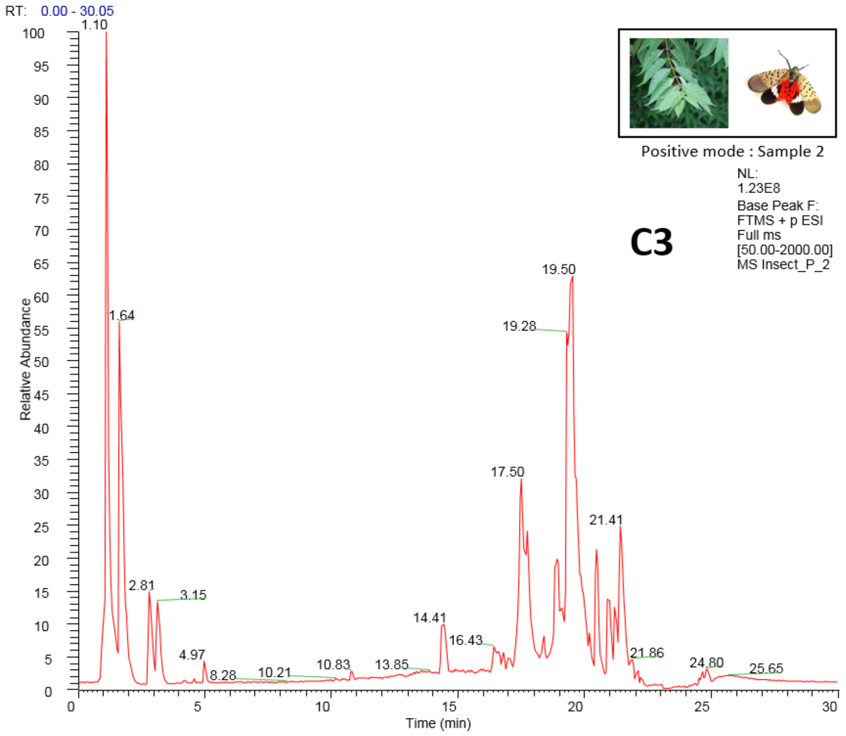
RT: 0.00 - 30.05
Positive mode : Sample 2
NL:
1.23E8
Base Peak F:
FTMS + p ESI
Full ms
[50.00-2000.00]
MS Insect_P_2
C3
Relative Abundance
Time (min)
1.10
1.64
2.81
3.15
4.97
8.28
10.21
10.83
13.85
14.41
16.43
17.50
19.28
19.50
21.41
21.86
24.80
25.65

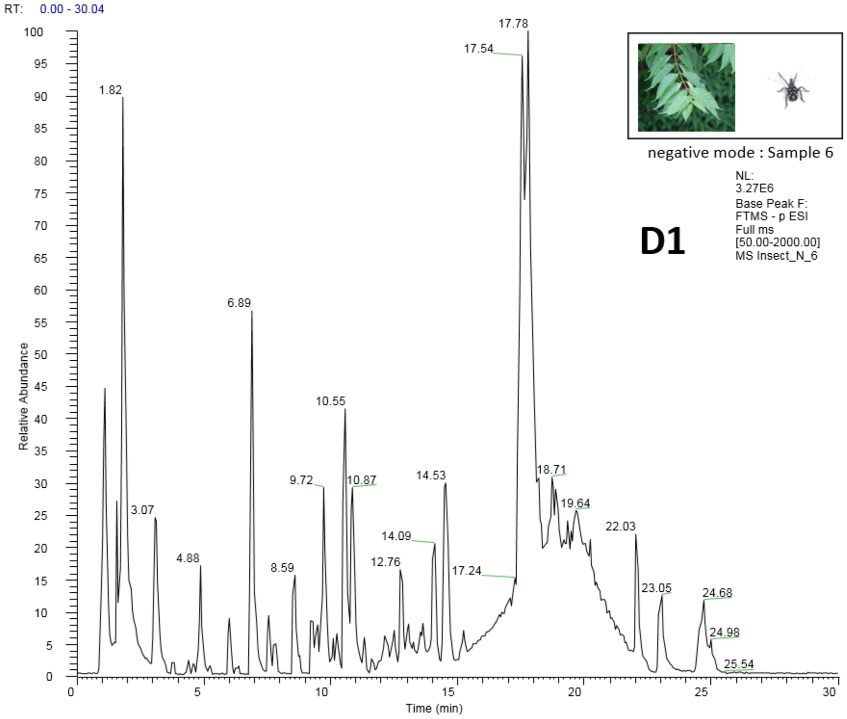
RT: 0.00 - 30.04
negative mode : Sample 6
NL:
3.27E6
Base Peak F:
FTMS - p ESI
Full ms
[50.00-2000.00]
MS Insect_N_6
D1
Relative Abundance
Time (min)
1.82
3.07
4.88
6.89
8.59
9.72
10.55
10.87
12.76
14.09
14.53
17.24
17.54
17.78
18.71
19.64
22.03
23.05
24.68
24.98
25.54

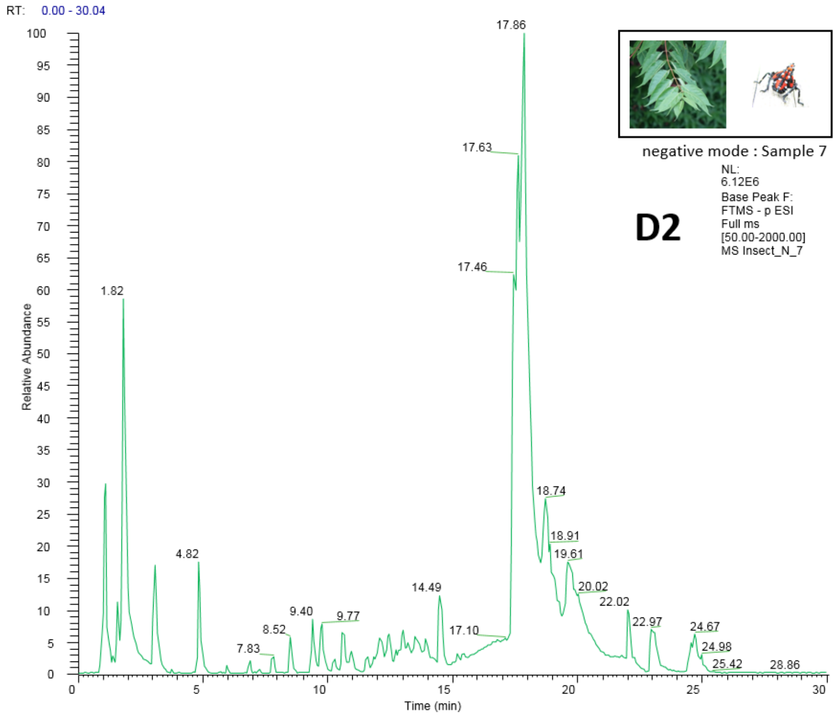

RT: 0.00 - 30.04
17.86
17.63
17.46
1.82
4.82
14.49
9.40
9.77
8.52
7.83
17.10
18.74
18.91
19.61
20.02
22.02
22.97
24.67
24.98
25.42
28.86
negative mode : Sample 7
NL:
6.12E6
Base Peak F:
FTMS - p ESI
Full ms
[50.00-2000.00]
MS Insect_N_7
D2
Relative Abundance
Time (min)

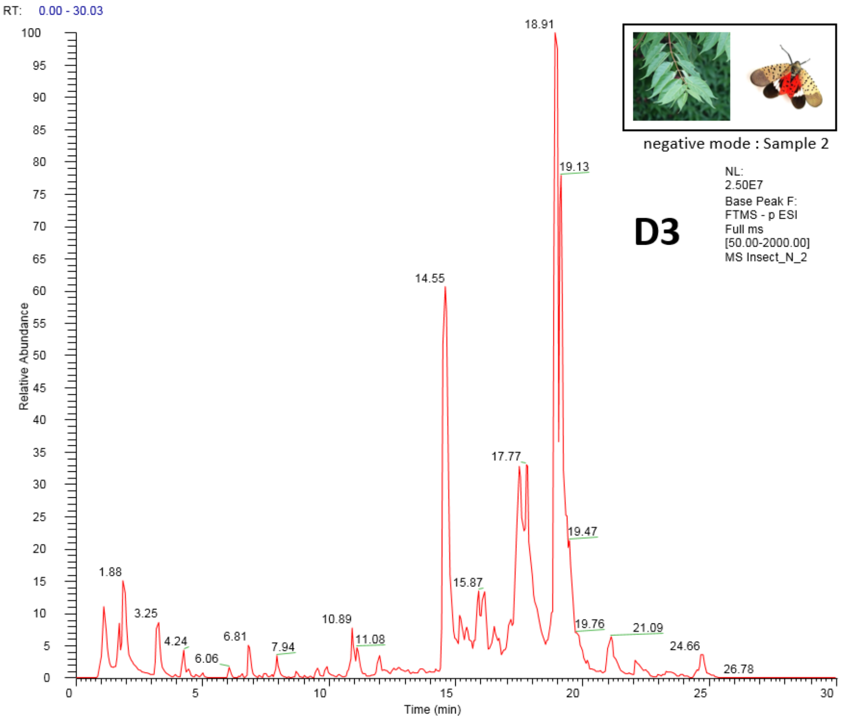

RT: 0.00 - 30.03
18.91
19.13
14.55
17.77
19.47
15.87
1.88
3.25
4.24
6.06
6.81
7.94
10.89
11.08
19.76
21.09
24.66
26.78
negative mode : Sample 2
NL:
2.50E7
Base Peak F:
FTMS - p ESI
Full ms
[50.00-2000.00]
MS Insect_N_2
D3
Relative Abundance
Time (min)

Fig S2. (previous pages) Mass spectrometry outputs in the positive and negative modes for the insect specimens from *Salix koreensis* and *Ailanthus altissima*. Fig. S2-A1: positive mode, *Salix koreensis*, instar 3; Fig. S2-A2: positive mode, *Salix koreensis*, instar 4; Fig. S2-A3: positive mode, *Salix koreensis*, adult; Fig. S2-B1: negative mode, *Salix koreensis*, instar 3; Fig. S2-B2: negative mode, *Salix koreensis*, instar 4; Fig. S2-B3: negative mode, *Salix koreensis*, adult; Fig. S2-C1: positive mode, *Ailanthus altissima*, instar 3; Fig. S2-C2: positive mode, *Ailanthus altissima*, instar 4; Fig. S2-C3: positive mode, *Ailanthus altissima*, adult; Fig. S2-D1: negative mode, *Ailanthus altissima*, instar 3; Fig. S2-D2: negative mode, *Ailanthus altissima*, instar 4; Fig. S2-D3: negative mode, *Ailanthus altissima*, adult.

PART 2) : Fig. S3 (this page and the following one). Results of the second set of mass spectrometry analyses ("Liquid chromatography triple quadrupole mass spectrometry ")

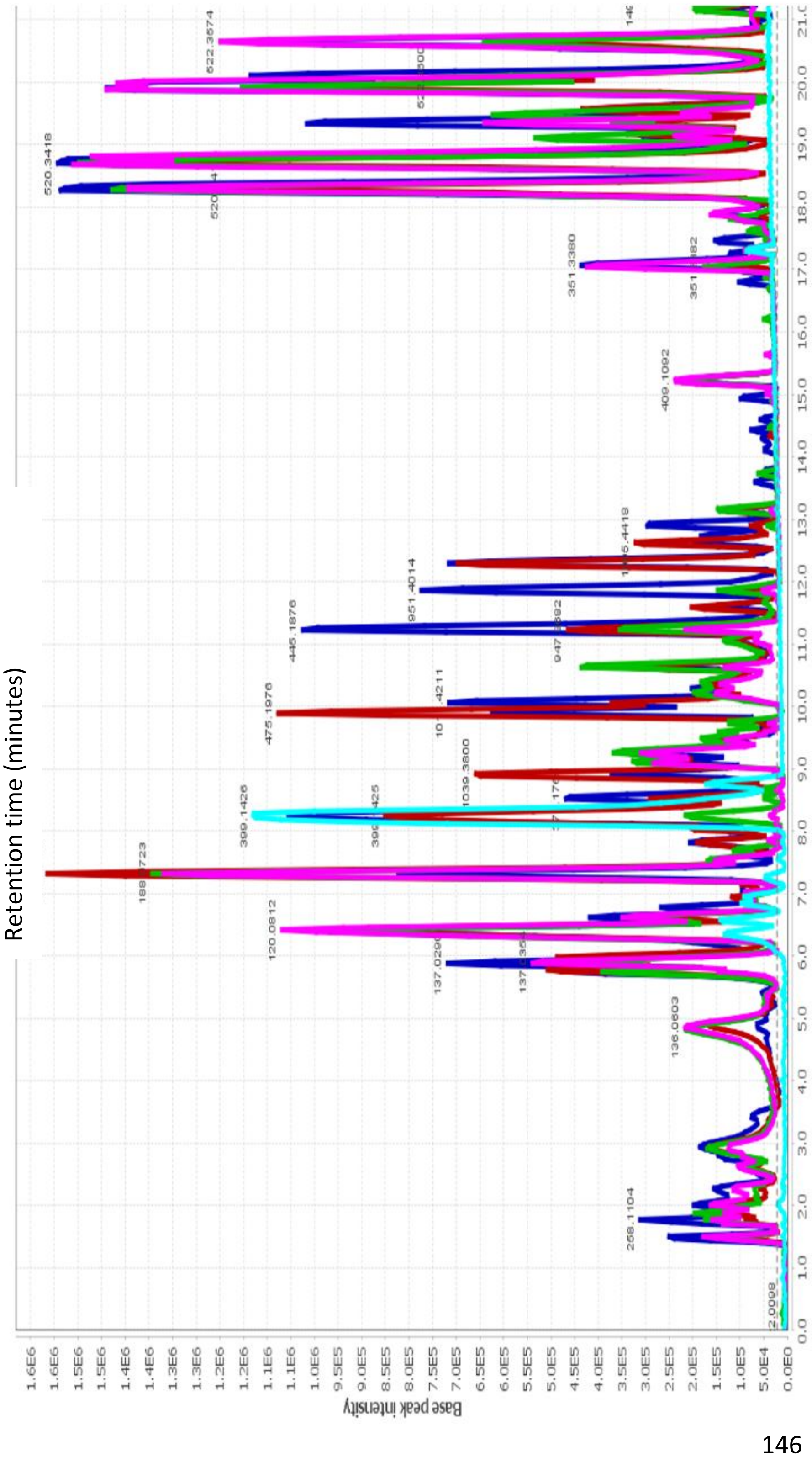

Fig. S3 (A) – Overlaid chromatograms for the first 21 minutes of retention time in the second set of mass spectrometry analyses comparing nymphs from the tree of heaven (red line), adults from the tree of heaven (blue line), nymphs from the persimmon tree (pink line) and adults from the persimmon tree (green line), along with the ailanthone standard reagent (sky blue line). Numbers at the peaks indicate m/z values. (Raw data files available upon request).

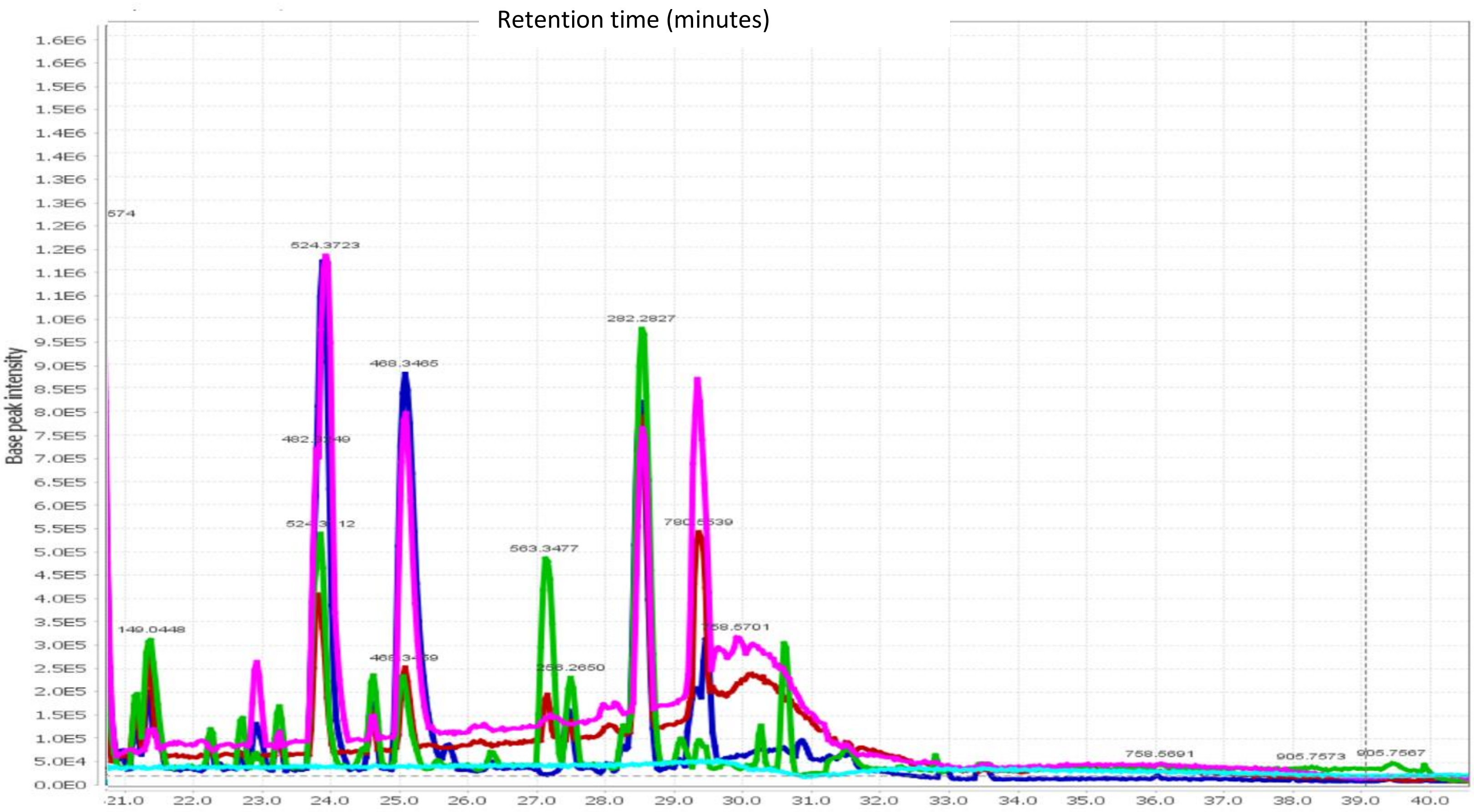

Fig. S3 (B) – Overlaid chromatograms for the first 21 minutes of retention time in the second set of mass spectrometry analyses comparing nymphs from the tree of heaven (red line), adults from the tree of heaven (blue line), nymphs from the persimmon tree (pink line) and adults from the persimmon tree (green line), along with the ailanthone standard reagent (sky blue line). Numbers at the peaks indicate m/z values. (Raw data files available upon request).

PART 3); Alternative defensive chemical compounds in Chinese populations

While our Korean population of spotted lantern-flies sequestered quassinoids, the population in China reported by Xue and Yuan (1996a, b) contained alkaloids yohimbine and ajmalicine. Therefore, we compared mass spectrometry results (see "*LCQ mass spectrometry* " in Methods of the main text) for adult spotted lanternflies collected on the tree of heaven with results for yohimbine and aimalicine standards. Standards were prepared from products purchased in SIGMA: Aijmalicine (41111 SIGMA, ≥98.0%, HPLC) and Yohimbine hydrochloride (Y3125 SIGMA, ≥98.0%, TLC, powder). We did not find clear peaks corresponding to either yohimbine or aimalicine (example in Fig. S6).

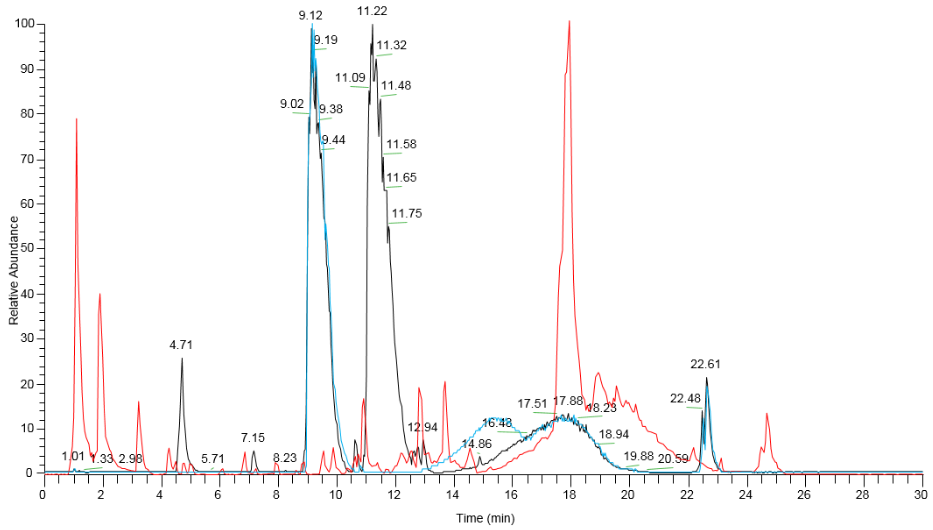

Fig S6. Example of mass spectrometry profiles (negative mode) showing that neither yohimbine nor aimalicine are present in the bodies of the spotted lanternflies in our population. Red – adult spotted lanternfly from the tree of heaven; Blue – yohimbine standard; Black – Yohimbine mixed with aimalicine.

Xue and Yuan (1996a, b) did not report the host plant species but it is possible that the insects metabolised or sequestered the alkaloids from local host plants. We hypothesize that these host plants may have belonged to the plant family Apocynaceae, which is also represented among *L. delicatula* host plants in Korea (Park et al. 2009). The family contains members known to produce yohimbine and ajmalicine (Raffauf & Flagler 1960, Liu, Cao, Yao & Xu 2013) and is common in tropical and subtropical regions (Endress & Bryuns 2000), which includes the native range of the spotted lantern-fly in China (Xiao 1991). This points to the possibility that the spotted lanternflies sequester various potentially unpalatable compounds from locally available plants. In this case, *L. delicatula* may be studied in the context of plasticity in sequestration of chemical defenses in an aposematic insect.

References

Endress, M. E., & Bruyns, P. V. (2000). A revised classification of the Apocynaceae sl. *The Botanical Review*, 66(1), 1-56.

Liu, L., Cao, J. X., Yao, Y. C., & Xu, S. P. (2013). Progress of pharmacological studies on alkaloids from Apocynaceae. *Journal of Asian natural products research*, 15(2), 166-184.

Park, J. D., Kim, M. Y., Lee, S. G., Shin, S. C., Kim, J. H., & Park, I. K. (2009). Biological characteristics of *Lycorma delicatula* and the control effects of some insecticides. *Korean journal of applied entomology*, 48(1), 53-57.

Raffauf, R. F., & Flagler, M. B. (1960). Alkaloids of the Apocynaceae. *Economic Botany*, 14(1), 37-55.

Xiao, G. G. (1991). *Forest insects of China*, 2nd edn. China Forestry Publishing House.

Xue, G., & Yuan, S. (1996a). Chemical constituents of the Lycorma delicatula. *Chinese Pharmaceuticcal Journal - Beijing*, 31, 651-654.

Xue, G., & Yuan, S. (1996b). Separation and preparation of indole alkaloids in *Lycorma delicatula* White. by HPLC. *China journal of Chinese materia medica*, 21(9), 554-555.
